# Temephos Resistance in *Aedes aegypti* in Colombia Compromises Dengue Vector Control

**DOI:** 10.1371/journal.pntd.0002438

**Published:** 2013-09-19

**Authors:** Nelson Grisales, Rodolphe Poupardin, Santiago Gomez, Idalyd Fonseca-Gonzalez, Hilary Ranson, Audrey Lenhart

**Affiliations:** 1 Department of Vector Biology, Liverpool School of Tropical Medicine, Liverpool, United Kingdom; 2 Grupo de Biología y Control de Enfermedades Infecciosas, Universidad de Antioquia, Medellín, Colombia; 3 Instituto de Biología, Universidad de Antioquia, Medellín, Colombia; 4 Entomology Branch, Division of Parasitic Diseases and Malaria, Center for Global Health, Centers for Disease Control and Prevention, Atlanta, Georgia, United States of America; Fundaçao Oswaldo Cruz, Brazil

## Abstract

**Background:**

Control and prevention of dengue relies heavily on the application of insecticides to control dengue vector mosquitoes. In Colombia, application of the larvicide temephos to the aquatic breeding sites of *Aedes aegypti* is a key part of the dengue control strategy. Resistance to temephos was recently detected in the dengue-endemic city of Cucuta, leading to questions about its efficacy as a control tool. Here, we characterize the underlying mechanisms and estimate the operational impact of this resistance.

**Methodology/Principal Findings:**

Larval bioassays of *Ae. aegypti* larvae from Cucuta determined the temephos LC_50_ to be 0.066 ppm (95% CI 0.06–0.074), approximately 15× higher than the value obtained from a susceptible laboratory colony. The efficacy of the field dose of temephos at killing this resistant Cucuta population was greatly reduced, with mortality rates <80% two weeks after application and <50% after 4 weeks. Neither biochemical assays nor partial sequencing of the *ace-1* gene implicated target site resistance as the primary resistance mechanism. Synergism assays and microarray analysis suggested that metabolic mechanisms were most likely responsible for the temephos resistance. Interestingly, although the greatest synergism was observed with the carboxylesterase inhibitor, DEF, the primary candidate genes from the microarray analysis, and confirmed by quantitative PCR, were cytochrome P450 oxidases, notably *CYP6N12*, *CYP6F3* and *CYP6M11*.

**Conclusions/Significance:**

In Colombia, resistance to temephos in *Ae. aegypti* compromises the duration of its effect as a vector control tool. Several candidate genes potentially responsible for metabolic resistance to temephos were identified. Given the limited number of insecticides that are approved for vector control, future chemical-based control strategies should take into account the mechanisms underlying the resistance to discern which insecticides would likely lead to the greatest control efficacy while minimizing further selection of resistant phenotypes.

## Author Summary

Dengue fever, caused by viruses transmitted by the *Aedes aegypti* mosquito, is an important threat to public health in many tropical and subtropical countries. In the absence of a vaccine or specific drug treatment, prevention and control of dengue transmission relies on interventions targeting vector mosquito populations. In the city of Cucuta, Colombia, the insecticide temephos was used for several decades to control *Ae. aegypti* larvae, until resistance was recently reported. In this study, the resistance to temephos in this population was quantified, and its impact on control activities estimated using simulated field trials. The mechanisms underlying the resistance were determined to be metabolic, with several key detoxification enzymes identified as potential candidates. This should be taken into account when devising future vector control and insecticide resistance management strategies in this region of Colombia.

## Introduction

Dengue fever is the most rapidly expanding arboviral disease in the world. Approximately 50 million infections occur in 100 countries annually [1], [2], and 60% of those are estimated to occur in the Americas [3]. In Colombia, dengue is considered a major public health problem, with approximately 25 million people at risk of infection. The primary vector of dengue, the *Aedes aegypti* mosquito, is found in more than 90% of the national territory [4].

*Ae. aegypti* is highly anthropophilic, with markedly endophilic and endophagic behaviors; these characteristics are directly related to its high efficiency as a disease vector [5], [6]. In the absence of a vaccine or effective therapeutic medications, vector control remains the only available strategy to control and prevent dengue transmission [6].

Many dengue vector control interventions target the immature stages of the mosquito, which breed in artificial containers in close proximity to human dwellings. The most widely used method for controlling immature *Ae. aegypti* is the periodic treatment of actual and potential breeding sites with chemical larvicides. The organophosphate (OP) insecticide temephos is commonly used to control immature dengue vectors due to its cost-effectiveness and community acceptance [5], [7], [8]. As a consequence of its widespread use, resistance to temephos in *Ae. aegypti* has been reported in many Latin American countries, including Brazil [9], Cuba [10], El Salvador [11], Argentina [12], Bolivia [13], Venezuela [14], Peru [15] and Colombia [16]. It is believed that the extent of temephos resistance is underestimated due to under-reporting and lack of surveillance [8].

Despite increasing reports of temephos resistance in *Ae. aegypti*, the molecular mechanisms underpinning it are not well-characterized. In several mosquito species of medical importance such as *Anopheles gambiae*, *Culex pipiens* and *Culex tritaeniorhynchus*, mutations on the acetylcholinesterase gene (*ace-1*) have been associated with OP resistance [16], [17], [18]. However, no mutations at this target site have been found related to OP resistance in *Ae. aegypti*. The three main enzyme families involved in xenobiotic detoxification in mosquitoes, glutathione S-transferases (GST), cytochrome P450 monooxygenases (CYP450) and carboxylesterases (CE) have been associated with temephos resistance in *Ae. aegypti* [19], with elevated CE activity most widely implicated. Recently, increased activity of the esterase “A4” in *Ae. aegypti* was partially characterized and strongly correlated with temephos resistance [20]; however, its genomic identity remains unknown.

Temephos is currently one of the most commonly used insecticides in Colombia [21]. In the densely populated, dengue endemic city of San Jose de Cucuta (‘Cucuta’), temephos was used for nearly 40 years as a routine *Ae. aegypti* control measure but applications ceased when resistance was recently detected. Despite the potential implications of this resistance for the efficacy of dengue vector control, neither the operational impact nor the mechanisms of temephos resistance have been characterized. In this study, we explore the mechanisms of temephos resistance in *Ae. aegypti* from Cucuta and estimate the impact of this resistance on the efficacy of temephos-based vector control operations.

## Methods

### Study site

Cucuta is a city located in the eastern range of the Andes mountains of Colombia (7°54′0″N, 72°30′0″W), at 320 meters above sea level and with an average temperature of 28°C. Since the municipal water supply is frequently interrupted, people typically store water in large ground level cement tanks, or in some cases, in plastic tanks on the roof. These containers provide abundant breeding sites for *Ae. aegypti*.

### *Ae. aegypti* collections

Verbal permission was obtained from householders to conduct entomological collections on their premises in March 2010. Oviposition traps (‘ovitraps’) were placed in 500 houses, while approximately 200 houses were visited for larval collections. The houses were located in five different areas of the city which were selected due to historically high levels of dengue transmission. Larval collections were made directly by removing larvae from household water storage tanks and other breeding sites, such as cans, bottles, tires, and miscellaneous discarded items, generally located in the patio area. They were taken to the insectaries at the Biologia y Control de Enfermedades Infecciosas group at the Universidad de Antioquia in Medellin, and reared under standard conditions (temperature: 28+/−1°C; relative humidity: 75+/−5%; photoperiod: 12 hours day/night). To increase larval numbers, approximately 500 ovitraps [22] were placed inside houses and in backyard/patio locations. After four days, the ovitraps were retrieved and checked for eggs. Positive traps were taken to the insectary where the eggs were hatched and the offspring were reared. All field samples were pooled to create the Cucuta strain.

Three insecticide-susceptible strains of *Ae. aegypti* were used as controls in this study. The New Orleans (NO) strain was originally collected in the namesake city located in Louisiana, United States. The Rockefeller (RCK) strain originated in Cuba nearly a century ago, while the Bora Bora (BB) strain was collected on its namesake island in French Polynesia in the 1960s [23].

### Insecticide susceptibility tests

Standard WHO larval bioassays were conducted to detect the level of susceptibility to temephos [24]. Each bioassay consisted of four replicates per insecticide concentration; each replicate used twenty late 3rd/early 4th instar larvae. Eight doses of temephos (Pestanal, analytic standard) ranging from 0.01 to 0.15 ppm were tested with both the Cucuta strain and a susceptible reference strain (NO). Mortality was recorded after 24 hours of exposure. LC_50_ values and confidence intervals were calculated using XLSTAT software (Addinsoft, Paris, France). Given that permethrin resistance had previously been reported in this population, standard WHO larval bioassays were also conducted using 6 concentrations of permethrin: 0.0075, 0.01, 0.02, 0.03, 0.04 and 0.05 ppm.

### Synergist bioassays

To assess the role of the three main detoxification enzyme families in temephos resistance, larvae were exposed to either diethyl-maleate (DEM), piperonyl-butoxide (PBO) or S,S,S-tributyl phosphorotrithioate (DEF) (Sigma-Aldrich) as inhibitors of GSTs, CYP450s and CEs, respectively. Standard temephos larval bioassays with three doses ranging from 0.05 ppm to 0.15 ppm were carried out with the addition of a specified concentration of synergist: either DEM at 1 ppm, PBO at 0.3 ppm or DEF at 0.5 ppm [25]. Each bioassay consisted of three replicates per insecticide concentration; each replicate used twenty late 3rd/early 4th instar larvae. The same assay was carried out using the NO strain as a control. Resistance ratios were calculated by dividing Cucuta values by NO values at LC_50_, and synergism ratios were calculated as the ratio between the LC_50_ obtained with each synergist and the LC_50_ obtained without synergists.

### Temephos efficacy assay

This assay was conducted in a semi-field environment at the University of Antioquia in Medellin, Colombia. Based on the methodologies proposed by Montella *et al.* and Lima *et al.* [26], [27], white plastic buckets were filled with 15 liters of tap water, and were kept outdoors, protected from direct rain and sunlight exposure. Mesh lids secured with elastic bands were used to prevent the introduction of wild mosquitoes or detritus into the buckets during the course of the experiment. A dose of 1 ppm of temephos (Abate, Fitogranos, Bogotá, Colombia), was used to treat each container, which is equivalent to the dose applied to breeding sites by vector control personnel. There were 2 experimental groups, each with three replicates: in Group 1, 3 liters of the temephos-treated water were replaced with fresh, untreated tap water twice a week, while in Group 2, the original temephos-treated water remained for the duration of the experiment without replacement. Twice a week over a 2-month period, 20 third instar larvae from the temephos-resistant Cucuta strain (F4 generation) were introduced into each container and mortality was recorded after 24 hours. Both dead and surviving larvae were removed from the containers after counting. Simultaneously, the same methodology was carried out using larvae from the RCK susceptible reference strain. Control containers without temephos were maintained under the same conditions for both experimental groups. Water temperature and pH were recorded twice a week, before each mortality recording.

### Biochemical assays

Activity levels of insensitive acetylcholinesterase (iAChE), glutathione S-transferases (GST), mixed function oxidases (MFO), α-esterases and β-esterases were tested in Cucuta larvae, with larvae from the NO strain used as a negative control. Procedures were based on mosquito-specific biochemical assay protocols reported elsewhere [28], [29], [30]. Briefly, 30 individual larvae were homogenized in 100 µL of 0.01 M potassium phosphate buffer (KPO_4_), ph 7.2, and then the volume was diluted to 2 ml, and 100 µl of each sample were transferred by triplicate to a 96-well microtiter plate. For the iAChE assay, 100 µl of acetylcholine iodide (ATCH) with propoxur and 100 µl of dithio-bis2-nitrobenzoic acid were added to each well; absorbance was recorded immediately (T_0_) and after 10 minutes (T_10_) in a Varioskan Flash Multimode Reader (Thermo Scientific, Delaware, USA) at a wavelength of 414 nm. As a positive control for elevated iAChE activity, the *Anopheles gambiae* AKRON strain (supplied by MR4, Manassas, Virginia, USA) was used.

For the GST assay, 100 µl of reduced glutathione and 100 µl of 1-chloro-2,4 – dinitrobenzene (CDNB) were added to each well. Absorbance readings were taken at T_0_ and T_10_ at a wavelength of 340 nm. For the MFO assay, 200 µl of tetramethyl-benzidine dihydrochloride (TMBZ) prepared in methanol and 0.25 M sodium acetate buffer were added to each well, followed by 25 µl of 3% hydrogen peroxide (H_2_O_2_). The microplate was incubated at room temperature for 10 minutes before reading at a wavelength of 620 nm. To detect α- and β-esterase activity, 100 µl of α-/β-naphthyl acetate were added to each well, followed by a 20 minute incubation at room temperature. 100 µl of dianizidine were then added, followed by a 4 minute incubation, and then absorbance was read at a wavelength of 540 nm.

To avoid bias due to natural variations in the size of the larvae, the total protein content of each sample was estimated. In triplicate, 200 µl of Bradford® reagent was added to 20 µl of homogenate (diluted to 100 µL by adding KPO_4_ buffer) and the microplate was read at 620 nm. A standard bovine serum albumin (Sigma) calibration curve was done for comparison. All replicates showing a coefficient of variation >0.20 were discarded.

### *Ace-1* sequencing

#### RNA extraction and cDNA synthesis

Larvae from the Cucuta strain surviving temephos exposure at a LC_90_ were categorized as resistant and stored in trizol after bioassay. RNA from two pools of 10 larvae was extracted using the TRIzol/chloroform RNA extraction method, according to the manufacturer (Invitrogen, Carlsbad, USA). RNA yields were assessed using a Nanodrop ND-1000 (Thermo Scientific). After treatment with DNase (Invitrogen), a reverse transcription using Oligo-dT20 (Invitrogen) and Superscript III (Invitrogen) was done. The resulting cDNA was used as PCR templates.

#### *Ace-1* cloning and sequencing

*Ace-1* exons 4–7 were selected for sequencing based on the presence of mutations associated with OP resistance in other mosquito species, specifically at positions G119S and F290V. Two external primers were employed in the PCR, while three pairs of internal primers were used for the sequencing (Table S1). The a*ce-1* PCR was carried out using Phusion® High-Fidelity DNA Polymerase (Thermo Scientific). For 30 cycles, denaturing, annealing and extension conditions were 94°C for 15 seconds, 55°C for 30 seconds and 68°C for 100 seconds, respectively.

PCR products were visualized on a 1% agarose gel and purified using a GeneJET Gel Extraction Kit (Fermentas). The *Ace-1* fragments were cloned (pJET 1.2/blunt Cloning Vector, Fermentas) and purified with Minipreps (GeneJET Plasmid Miniprep Kit, Fermentas). Sequencing was performed by Macrogen (Amsterdam, the Netherlands).

### Genome-wide transcriptomic analysis

#### RNA extraction and labeled cRNA synthesis

RNA was extracted using the Arcturus Picopure RNA Extraction Kit® from pools of twenty larvae from 5 mosquito groups: 1) F2 generation of the Cucuta strain, unexposed to insecticide (CucU), 2) F2 generation of the Cucuta strain surviving LC_60_ of temephos (CucR), 3) F2 generation of the Cucuta strain surviving exposure to 0.04 ppm of permethrin (CucP), 4) NO reference strain unexposed to insecticide and 5) BB reference strain unexposed to insecticide. Because the temephos used to expose the larvae was diluted in ethanol and to minimize any non-insecticide related responses, CucU, NO and BB larvae were exposed to 1% ethanol for 24 hours prior to RNA extraction. 100 ng of each RNA sample were amplified and labeled using the low input Quick Amp Labeling Kit for 2 colours (Agilent Technologies). Each sample was labeled with Cy-3 and Cy-5 dyes in different tubes.

#### Hybridizations

After labeling, cRNA was purified using Qiagen RNeasy minispin columns (Qiagen, Hilden, Germany). Quality and quantity of RNA were assessed using a Nanodrop ND-1000 (Thermo Scientific, Delaware, USA) and an Agilent 2100 Bioanalyzer (Agilent Technologies, California, USA). Samples were analyzed using the 15K Agilent *Aedes* microarray chip (ArrayExpress accession number A-MEXP-1966), which contains probes for more than 14,320 *Ae. aegypti* transcripts. The CucR samples were competitively hybridized to either CucU, BB, NO or CucP. Three biological replicates were used with respective dye swapping in all experiments with the exception of CucR vs CucU in which only two biological replicates were used. Hybridization was performed over 17 hours at 65°C and 10 RPM. The microarray slides were then washed using the Agilent Microarray Hybridization Kit (Agilent Technologies), following the manufacturer's protocol.

#### Data acquisition and analysis

Microarrays were scanned using an Agilent G2205B microarray scanner (Agilent Technologies). The Agilent Feature Extraction software (Agilent Technologies) was employed for spot finding and signal quantification for both Cy-3 and Cy-5 dyes. Data normalization and statistical analyses were carried out using Genespring GX software (Agilent Technologies). A Student's t-test with a baseline value of 1 (same transcription level) and Benjamini and Hochbergs' multiple test correction was used to assess the over- or under-transcription of genes. Transcripts showing a 2-fold positive or negative change and a corrected P-value<0.01 were considered differentially transcribed between the experimental groups. In the CucR vs. CucU comparison, very few genes were differentially transcribed using these criteria so the P-value cutoff was increased to 0.02 and the fold change decreased to 1.5.

#### Quantitative PCR

To validate the microarray results, 4 genes were selected from the resulting microarray candidate gene list for quantitative real-time PCR analysis: *CYP6F3*, *CYP6N12*, *CYP6M11* and acetyl coA synthetase (AAEL015010). The qPCR primers were designed based on sequences retrieved from VectorBase. cDNA was obtained from larvae from the F4 generation of the Cucuta strain exposed to temephos under the same conditions as described for the microarray experiments using the reverse transcriptase Superscript® III (Invitrogen, Carlsbad, CA, USA). Each qPCR reaction of 25 µl contained 5 µl of cDNA, 0.3 M of each specific primer (Table S2) and 12.5 µl of Fast Start SYBR Green Master Mix. To verify the specificity of the primers, melting curve analyses were conducted. Standard curves were produced from cDNA serial dilutions in order to avoid bias due to differences in PCR efficiency. To determine the fold change in each selected candidate gene, the 60S ribosomal protein L8 (AAEL000987) and the 40S ribosomal protein S7 (AAEL009496) were selected as references. The relative expression of each gene was calculated using the 2^−ΔΔCT^ method [31]. Statistical analyses and normalization were carried out using the Relative Expression Software Tool (REST) [32].

## Results

### Bioassays and synergist bioassays

The LC_50_ of temephos for the Cucuta strain of *Ae. aegypti* was 0.066 ppm (95% CI 0.06–0.074), approximately 15× higher than the value for the susceptible NO strain (0.0043; 95% CI 0.004–0.005). The LC_95_ of the Cucuta strain was 0.18 ppm (95% CI 0.15–0.23), with a resistance ratio (RR) of 14 relative to the NO strain.

Addition of the synergist DEF increased temephos susceptibility by approximately 36× in the Cucuta strain and by 7× in the New Orleans strain (Table 1). DEM resulted in a small decrease in the LC_50_ in the NO strain only. No effect of PBO was observed in either strain (Table 1).

**Table 1:** Susceptibility of *Ae. aegypti* larvae to temephos with and without synergists.

Strain	Treatment	LC_50_ (ppm) with 95% confidence Intervals	RR_50_	SR_50_
Cucuta	No synergist	0.0697	11.85	
		(0.062–0.0768)		
	DEM	0.0656	15.93	1.06
		(0.0595–0.0712)		
	PBO	0.0558	9.92	1.25
		(0.0463–0.063)		
	DEF	0.0019	2.43	36.28
		(0.0017–0.0022)+		
New Orleans	No synergist	0.0059		
		(0.0053–0.0066)		
	DEM	0.0041		1.43
		(0.0035–0.0049)+		
	PBO	0.0056		1.05
		(0.0049–0.0065)		
	DEF	0.0008		7.45
		(0.0007–0.0009)+		

+ Significant difference.

LC_50_: Lethal concentration at which 50% of the population is killed.

RR_50_: Resistance ratio at LC_50_, calculated as the ratio between Cucuta and NO LC_50_.

SR_50_: Synergism ratio at a LC_50_, calculated for each strain as the ratio between the LC_50_ of the treatment without synergist and the LC_50_ using each synergist.

DEM (diethyl maleate); PBO (piperonyl butoxide, 5-((2-(2-butoxyethoxy) ethoxy) methyl)-6-propyl-1,3-benzodiox-ole); DEF (S,S,S-tributyl phosphorotrithioate.

Larval bioassays using permethrin indicated that the Cucuta strain was also resistant to this insecticide, with an LC_50_ of 0.017 ppm (95% CI 0.015–0.019) and a RR_50_ of 16 relative to NO.

### Temephos efficacy assay

**Figure 1:**
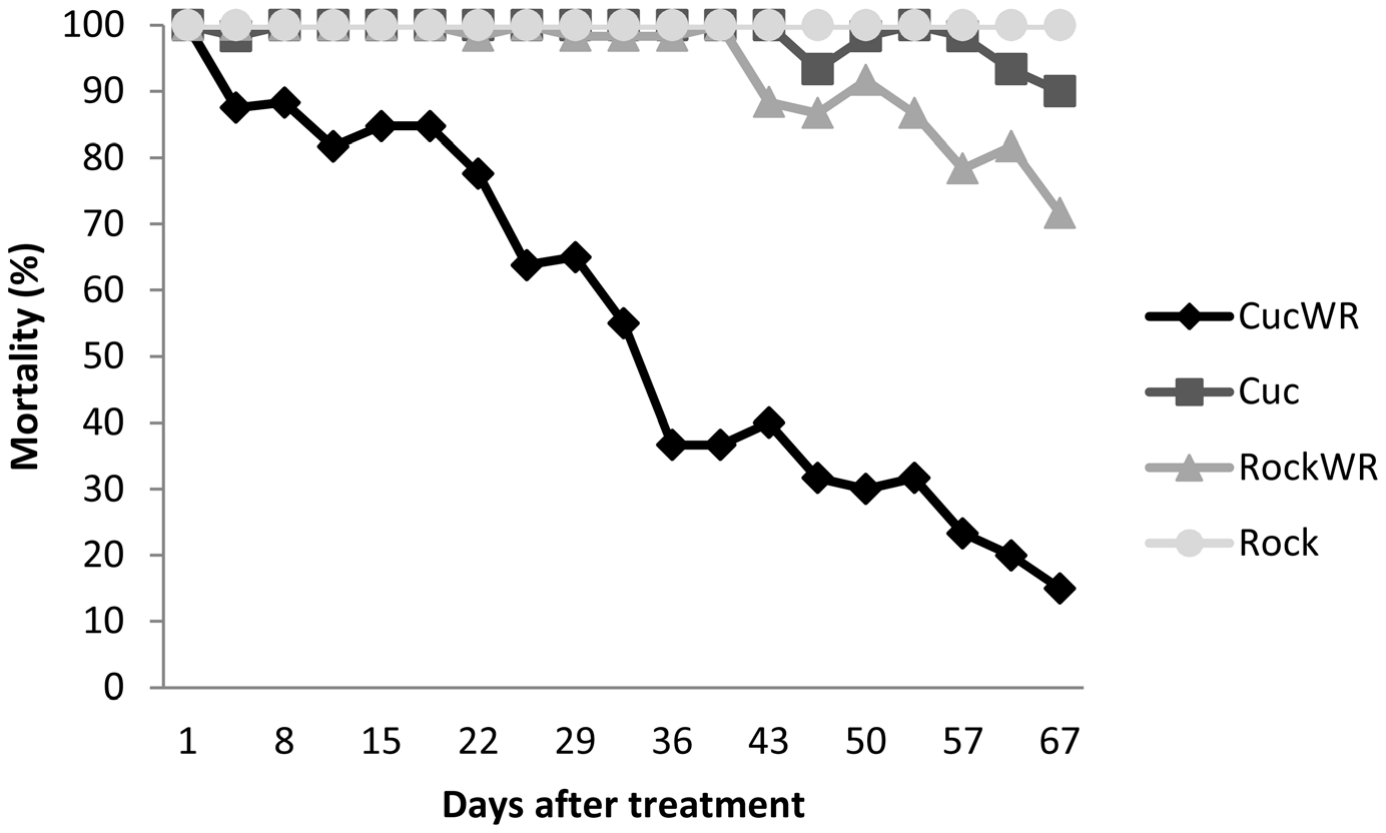
Temephos efficacy bioassay results. Percent mortality of Cucuta and RCK *Ae. aegypti* strains at different time points after application of temephos (1 ppm), with and without water renewal. CucWR/RockWR: assays of Cucuta and RCK strains with water renewal. Cuc/Rock: assays without water renewal. 95% confidence intervals are shown.

Temephos applied at the concentration used in routine vector control activities remained effective against both the RCK reference strain and the Cucuta strain for 8 weeks, provided the water was not replaced. With water replacement, over 20% of the Cucuta mosquitoes were surviving in treated containers by 4 weeks post-treatment, and nearly 80% were surviving after 2 months (Figure 1). In contrast, 100% mortality of the susceptible RCK larvae was maintained up to week six with water replacement. Water temperature for all containers ranged between 21°C and 25°C, and pH ranged between 8.0 and 8.6 (except during the second week when it briefly decreased to 7.4).

### Biochemical assays

None of the enzyme families in the Cucuta strain showed enhanced activity with the model substrates used, when compared with the NO strain. Similarly, there was no evidence of AChE insensitivity, with both the Cucuta and NO strains being equally inhibited by propoxur (Supplementary information, Figure S1).

### *Ace-1* partial sequencing

A 1614 bp fragment of the *ace-1* gene was sequenced from two pools of 10 larvae from the Cucuta strain. No amino acid polymorphisms were identified within the strain and only a single synonymous mutation was detected (position 1423, CTA to TTA) when compared to the reference sequence in Vectorbase (AAEL000511).

### Microarrays

**Figure 2:**
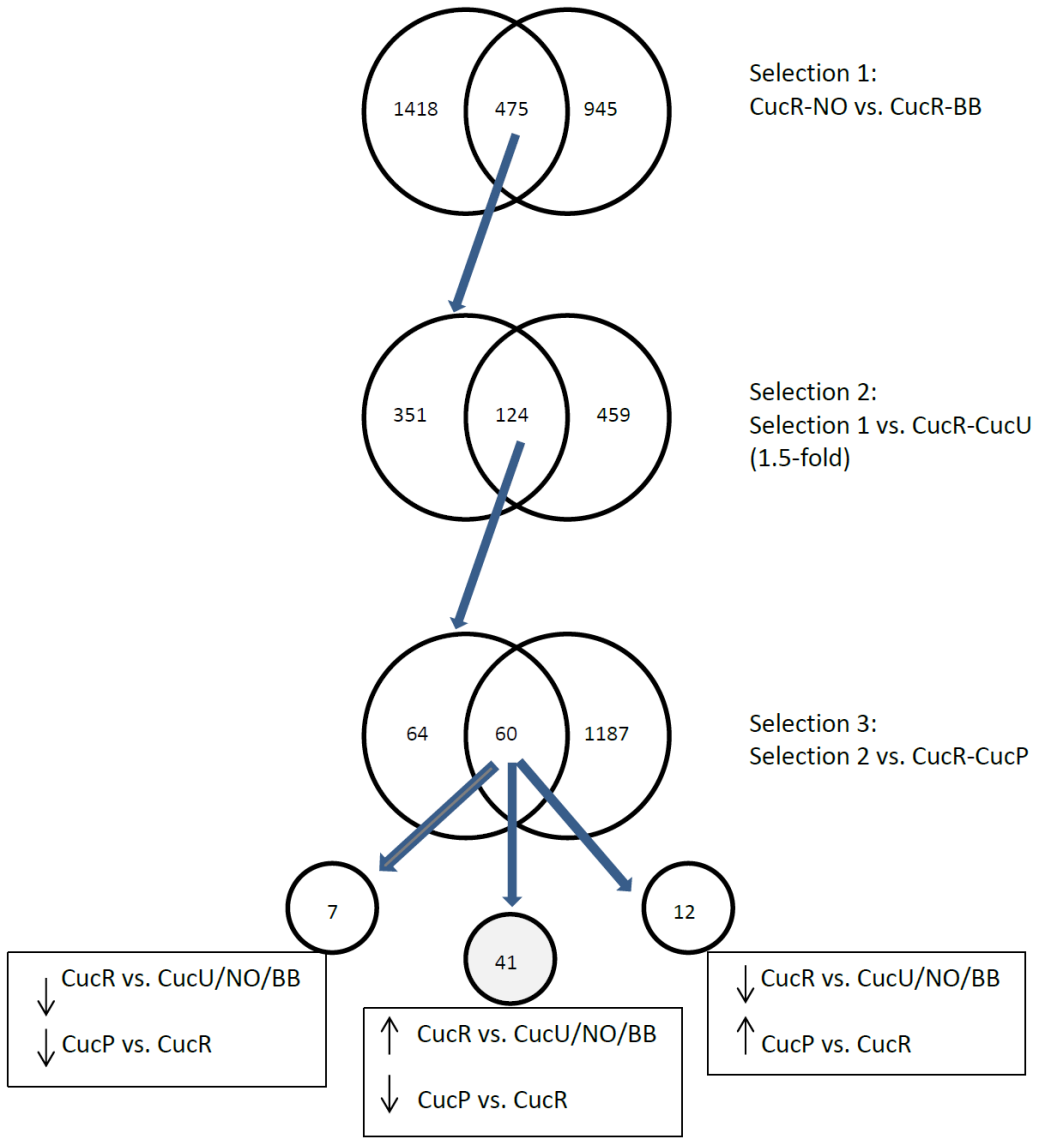
Flowchart for temephos resistance candidate gene selection based on microarray results. In selection 1, genes differentially regulated in both CucR-NO and CucR-BB microarrays were selected. These genes were compared with differentially transcribed genes from the CucR-CucU comparison, and the subset differentially expressed in both formed selection 2. This list was then compared with the differentially transcribed genes from CucP-CucU to select the genes that were under-transcribed in CucP vs. CucR microarray, resulting in 41 candidate genes.

To select candidate genes related to temephos resistance, genes significantly differentially expressed in each experiment were filtered as shown in Figure 2. Firstly, only genes that were differentially transcribed in both CucR-NO and CucR-BB microarrays were selected (Table S3a). This resulted in a list of genes differentially transcribed between Cucuta and both geographically distinct reference strains, thus reducing the bias introduced by differences in geographic origin of the reference strains. Then, this list was cross-referenced with the differentially transcribed genes resulting from CucR-CucU microarray. This step removed any genes that did not show differential expression between the unexposed Cucuta population and those surviving the temephos LC_50_ in an attempt to select for genes contributing to the temephos-resistant phenotype. The resulting gene list (Table S3b) contained 124 probes, 63 of which were upregulated in the CucR population in all 3 comparisons. This list was then further reduced by filtering out any probes that were more highly expressed in Cucuta mosquitoes surviving permethrin exposure than those surviving temephos exposure, resulting in a final list of 41 upregulated candidate genes associated with temephos resistance (Figure 2; Table 2).

**Table 2:** Temephos resistance candidate genes ranked according to the fold change in expression between CucR and CucU, with detoxification genes shown in bold.

		Fold change			
Systematic name	Description	CucR-NO	CucR-BB	CucR-CucU	CucP-CucR
AAEL011230-RA	Chymotrypsin, putative	87.14	28.37	30.21	−122.93
AAEL011167-RA	Cathepsin l	5.44	17.50	13.21	−4.32
AAEL001418-RA	Conserved hypothetical protein	26.24	4.70	13.00	−28.75
AAEL003076-RA	Glucosyl/glucuronosyl transferase	13.65	16.09	10.38	−22.37
AAEL015657-RA	Conserved hypothetical protein	14.54	13.51	8.14	−10.77
AAEL013263-RA	High affinity copper transporter, putative	4.41	9.73	6.73	−11.13
AAEL014843-RA	Heat shock protein	11.50	6.21	5.87	−3.21
AAEL011175-RA	Alkaline phosphatase	6.18	12.77	5.70	−7.80
AAEL000813-RA	Dimethylaniline monooxygenase	3.23	2.54	5.39	−6.71
AAEL015010-RA	Acetyl-coa synthetase	7.12	5.27	5.23	−5.23
AAEL015070-RA	Alkaline phosphatase	5.08	13.50	5.09	−6.85
AAEL006820-RA	Lipid storage droplets surface binding protein 2 (lsd2)	4.72	4.03	4.78	−2.49
AAEL001194-RA	Fatty acid synthase	5.47	3.23	4.14	−4.55
AAEL011704-RA	Heat shock protein	8.23	2.20	4.13	−3.05
AAEL007799-RA	Regulator of chromosome condensation	3.74	2.21	3.46	−2.88
AAEL007799-RB	Regulator of chromosome condensation	3.74	2.27	3.46	−2.83
AAEL004455-RA	Conserved hypothetical protein	3.62	2.16	3.12	−2.72
AAEL008651-RA	Conserved hypothetical protein	3.45	28.44	3.12	−3.97
AAEL008844-RA	Calcium-binding protein, putative	2.41	4.62	3.08	−5.07
AAEL008723-RA	Conserved hypothetical protein	4.36	2.01	2.92	−2.62
AAEL005045-RA	ATP-dependent bile acid permease	3.38	2.61	2.89	−7.49
AAEL000520-RA	Conserved hypothetical protein	5.77	2.95	2.58	−3.46
AAEL012185-RA	Ribosome biogenesis regulatory protein	2.93	2.77	2.58	−2.46
AAEL010118-RA	Kelch repeat protein	3.20	2.03	2.57	−2.29
AAEL012100-RA	Conserved hypothetical protein	3.18	3.11	2.43	−2.47
AAEL013406-RA	Venom allergen	7.90	4.23	2.42	−9.90
AAEL005991-RA	Tricarboxylate transport protein	2.21	4.37	2.38	−3.10
AAEL003654-RA	Conserved hypothetical protein	3.41	2.19	2.38	−2.16
AAEL009124-RA	**Cytochrome P450 CYP6N12**	3.98	4.40	2.34	−4.77
AAEL009127-RA	**Cytochrome P450 CYP6M11**	3.82	3.13	2.32	−2.25
AAEL007271-RA	Basic helix-loop-helix zip transcription factor	4.97	4.11	2.30	−3.41
AAEL012866-RA	Conserved hypothetical protein	3.88	4.38	2.29	−3.31
AAEL008281-RA	Conserved hypohetical protein	2.73	3.37	2.28	−5.23
AAEL003949-RA	Conserved hypothetical protein	2.50	2.55	2.26	−2.06
AAEL008124-RA	Possible RNA methyltransferase, putative	2.92	2.55	2.25	−2.01
AAEL000300-RA	WD-repeat protein	2.71	2.09	2.24	−2.08
AAEL013946-RA	Conserved hypothetical protein	3.88	5.35	2.17	−2.61
AAEL004063-RA	WD-repeat protein	2.90	2.50	2.08	−2.07
AAEL002913-RA	Peroxisomal membrane protein 70 abcd3	4.41	2.47	1.74	−2.39
AAEL014684-RA	**Cytochrome P450 CYP6F3**	3.35	7.22	1.71	−4.68
AAEL001813-RA	Sodium/solute symporter	3.08	2.70	1.66	−2.11

P-values and corrected P-values are shown in Table S3c.

The most over-transcribed gene in the CucR population was a putative chymotrypsin (AAEL011230-RA), with a 30-fold positive change when compared with CucU larvae. An UDP-glucosyl/glucuronosyl transferase (AAEL003076-RA) was also highly over-transcribed in this population. Three detoxification genes belonging to the CYP6 subfamily were also present on the temephos resistance candidate list: *CYP6N12*, *CYP6F3* and *CYP6M11* (Table 2). Microarray data were submitted to ArrayExpress (accession number E-MTAB-1682).

### Quantitative PCR

Three of the four genes selected for validation, *CYP6N12*, *CYP6F3* and the acetyl coA synthetase, showed similar fold changes by qPCR and microarray (Table 3). However, the over expression of *CYP6M11* was not confirmed by qPCR. A correlation analysis between the qPCR and microarray data yielded a R^2^ value of 0.31.

**Table 3:** Quantitative PCR and microarray results for four temephos resistance candidate genes.

		qPCR			Microarray	
Transcript	Strain	Fold change	2^−ΔΔCT^ LCI	2^−ΔΔCT^ UCI	Fold change	Adjusted P-value
CYP6N12	New Orleans	1.00	0.8	1.2		
	CucU	1.20	0.75	1.66	1.64	7.40E-02
	CucR	**3.94**	3.21	4.68	**3.98**	1.64E-05
CYP6F3	New Orleans	1.00	0.71	1.29		
	CucU	1.57	1.2	1.93	1.92	3.07E-03
	CucR	**3.66**	2.9	4.41	**3.35**	3.91E-05
CYP6M11	New Orleans	1.00	0.83	1.17		
	CucU	0.83	0.72	0.94	1.68	4.93E-03
	CucR	**1.04**	0.82	1.25	**3.82**	5.47E-05
Acetyl coA synthetase	New Orleans	1.00	0.91	1.09		
	CucU	2.33	1.9	2.77	1.32	1.41E-01
	CucR	**3.66**	1.45	5.88	**7.12**	8.49E-05

CucU: Cucuta larvae not exposed to temephos.

CucR: Cucuta larvae resistant to temephos.

## Discussion

In Colombia, dengue transmission is a major public health problem which has led to ongoing efforts to prevent and control dengue epidemics. As part of this effort, the National Network for Surveillance of Insecticide Resistance was created, and widespread screening of *Ae. aegypti* susceptibility was carried out in 2005–2008 across dengue endemic regions. Moderate to high levels of resistance were reported for all four major insecticide classes across the country [21], [33].

The principal intervention to control *Ae. aegypti* in Colombia is the application of temephos (Abate sand-core granules) at a concentration of 1 ppm to domestic and peridomestic water storage containers [34], as recommended by the WHO [5]. Resistance to temephos in *Ae. aegypti* has been previously reported in Colombia [21], [35]. In contrast with the findings presented here, one of these studies [21] detected elevated MFO and esterase activity in temephos resistant populations. The present study determined that *Ae. aegypti* from Cucuta were able to survive 15× higher doses of temephos than a standard susceptible strain.

Insecticide resistance can potentially compromise vector control measures. It has been reported that temephos resistance can affect the efficiency of this insecticide under both field and semi-field conditions [36], [37]. Insecticide bioassays with water renewal emulate the routine water replacement carried out in zones where *Ae. aegypti* breeding sites are intra- or peri-domestic water storage containers, offering a better approximation of the impact insecticide resistance may have on vector control measures [38]. The finding that, after approximately one month, the majority of Cucuta larvae survived this simulated field trial, suggests that the residual effect of routine control measures is compromised by the high level of resistance. Similar efficacy losses have been reported elsewhere in temephos resistant *Ae. aegypti* [26], [27], [38], [39].

Semi-field or laboratory bioassays can only detect resistance when it is already present in high frequencies in a vector population. Detecting resistance at an early stage could improve vector control efficacy by triggering the implementation of alternative control strategies pre-emptively, before resistance is present at high frequencies. In order to design appropriate diagnostic tools that can detect incipient resistance, the molecular mechanisms underlying resistant phenotypes must be characterized.

The most recognized OP target site resistance mechanism is insensitive acetylcholinesterase (iAChE). Although mutations on the gene encoding this enzyme (*ace-1*) have been associated with OP resistance in *Culex pipiens*, *Culex tritaeniorhynchus*, *Anopheles gambiae* and *Anopheles albimanus* [16], [17], [18], [40], this has not yet been observed in *Ae. aegypti*. It has been hypothesized that the absence of these mutations in this species is because some of the most common mutations, such as G119S, are unlikely to occur spontaneously [41]. The Cucuta strain did not exhibit iAChE and no amino acid changes on the *ace-1* gene were detected in temephos resistant mosquitoes. This is consistent with other findings that suggest that target-site resistance plays only a minor role in temephos resistance for *Ae. aegypti* [30].

Synergist bioassays suggested that carboxylesterases were potentially responsible for temephos resistance in the Cucuta strain. However, the biochemical assays did not detect any elevation in α- or β-esterase activity in the resistant population. There are several possible explanations for this apparent contradiction. The biochemical assays used model substrates which may not be recognized by all members of the CE family. In other insects, increased esterase activity has been associated with an amino acid alteration in a particular α-esterase [42], [43], [44] which is actually associated with a decrease in activity against a model substrate. Alternatively, the synergistic activity of DEF may be unrelated to its role as a CE inhibitor. To obtain a more comprehensive picture of specific genes involved in insecticide resistance, a transcriptional analysis was performed. This approach makes no assumption about the mechanisms involved but does rely on detecting changes in gene expression, and hence would not detect resistance mechanisms that resulted from an increased affinity of an enzyme for the insecticide, for example.

Two different susceptible reference strains, NO and BB, were used to minimize the genetic variation due to biological differences between strains. After a stringent analytical pipeline (Figure 2), 41 genes were identified that met the following criteria: 1. expressed at higher levels in the Cucuta population than in both the susceptible lab strains, 2. expressed at higher levels in Cucuta mosquitoes that had survived temephos exposure than in those not exposed to temephos and 3. expressed at higher levels in Cucuta mosquitoes surviving temephos exposure than in Cucuta mosquitoes surviving permethrin exposure. This final step was included as the Cucuta population was found to be resistant to both insecticides, but in this study we were particularly interested in those genes responsible for temephos resistance. It is recognized, however, that this step will have filtered out any genes that may be involved in cross resistance to both insecticides.

The final candidate list did not contain any CEs, which is in contrast with the extensive body of literature that closely relates OP resistance with this class of detoxifying enzymes [45], but in agreement with the results from the biochemical assays which did not support a role for elevated esterase activity in conferring the resistant phenotype. However, the possibility that temephos resistance is related to amino-acid substitutions on specific esterase genes, as has been previously reported for several insecticides in other dipteran species [43], [46], [47], [48], cannot be discounted by the microarray results.

Three gene members of the CYP6 P450 enzyme sub-family were found related to temephos resistance in this study. *CYP6M11* has been reported previously as induced in *Ae. aegypti* in response to xenobiotics [49], permethrin selection [50] and in larvae [51] and adults [19] of temephos-resistant strains. Although this gene was found to be upregulated in the microarray analysis, the over expression of this P450 could not be confirmed by qPCR. The genes *CYP6N12* and *CYP6F3*, identified as temephos resistance candidates in the current study, have previously been associated with resistance to the neonicotinoid imidacloprid and permethrin [50], [52], [53]; *CYP6N12* was also associated with tolerance to the polycyclic aromatic hydrocarbon (PAH) fluoranthene [53] and temephos [54].

It has previously been suggested that the conjugation of xenobiotics with glucose is an important detoxification pathway in insects [55]. Although *UDPGT*s have been described in some medically and agriculturally important insects as allelochemical detoxifiers [56], [57], they have only been demonstrated to be involved in insecticide resistance once [56]. Recently, high levels of *UDPGT* over-expression in the metabolic response of *Ae. aegypti* larvae to permethrin have been reported [50]. In the present study, one *UDPGT* (AAEL003076-RA) was associated with temephos resistance, suggesting that further studies are warranted on this transferase gene family to confirm its role in insecticide detoxification.

Serine proteases are a group of well-studied enzymes responsible for a variety of functions such as digestion, oogenesis, immune response, blood coagulation and metamorphosis [58], [59], [60]. In the present study, a chymotrypsin (AAEL011230-RA) was the most over transcribed gene in the temephos resistant population (30.2-fold difference between temephos survivors and non-exposed Cucuta larvae, and 87.1-fold difference between temephos survivors and NO). Although it has been reported previously that trypsins and chymotrypsins from *Culex pipiens pallens* are able to metabolize the pyrethroid deltamethrin [61], [62], there is no evidence so far to confirm that these enzymes can metabolize temephos. Organophosphate insecticides are also known to inhibit certain serine proteases, including chymotrypsins [63]. Functional characterization is needed to clarify the role of the chymotrypsin reported here in temephos resistance or in temephos-permethrin cross resistance.

The application of the organophosphate temephos to breeding sites is a pillar of *Ae. aegypti* immature control worldwide. However, its widespread, long-term use has led to the emergence of resistance in different parts of the world. Our findings demonstrate that a high level of temephos resistance significantly impacts the performance of this insecticide by reducing its residual efficacy by more than half, which in turn impacts vector control efficiency. As such, it is critical to develop tools that can detect resistance at its earliest stages of development, before resistance reaches levels at which control efficacy is compromised. The development of such tools requires a detailed understanding of the molecular basis and mechanisms underpinning resistance to insecticides. The results of the present study provide a comprehensive analysis of temephos resistance in *Ae. aegypti* from Cucuta, Colombia, and provide novel insights into the mechanisms underlying temephos resistance in this important disease vector. Through deeper understandings of the interactions between genes responsible for resistance to temephos and other insecticide groups, vector control programs can design control strategies that minimize the selection of resistant phenotypes and maintain vector control efficacy in the long term. In the case of Cucuta, the public health authorities have begun implementing alternative larval control strategies, including biological control (using small fish) and the application of pyriproxyfen (a juvenile hormone analogue) to breeding sites. Ongoing monitoring of temephos resistance will yield useful information about how the large scale deployment of these alternative strategies affects temephos resistance levels and its underlying mechanisms over time.

## Supporting Information

Figure S1 Box plots of corrected absorbance (nm) for enzyme activity, as measured by biochemical assays. A: Alpha-esterases; B: Beta-esterases; C: Mixed function oxidases; D: Glutathione S-transferases; E: Insensitive acetylcholinesterase.
(TIF)

Table S1 Summary of primer sequences for *ace-1* PCR and sequencing. Ace1-For and Ace1-Rev were the external primers used in the PCR. All other primers were used only for sequencing.
(RTF)

Table S2 Summary of primer sequences for quantitative real-time PCR.
(RTF)

Table S3 a Summary of temephos resistance candidate genes resulting from the first microarray data filter, ‘Selection 1’. **b**. Summary of temephos resistance candidate genes resulting from the second microarray data filter, ‘Selection 2.’ **c**. Summary of temephos resistance candidate genes resulting from the third microarray data filter, ‘Selection 3.’
(XLSX)
