# Supplementary figures and images for: Temephos Resistance in Aedes aegypti in Colombia Compromises Dengue Vector Control
Source: PLoS Negl Trop Dis. 2013 Sep 19;7(9):e2438. doi: 10.1371/journal.pntd.0002438 (PMC3777894; doi:10.1371/journal.pntd.0002438)

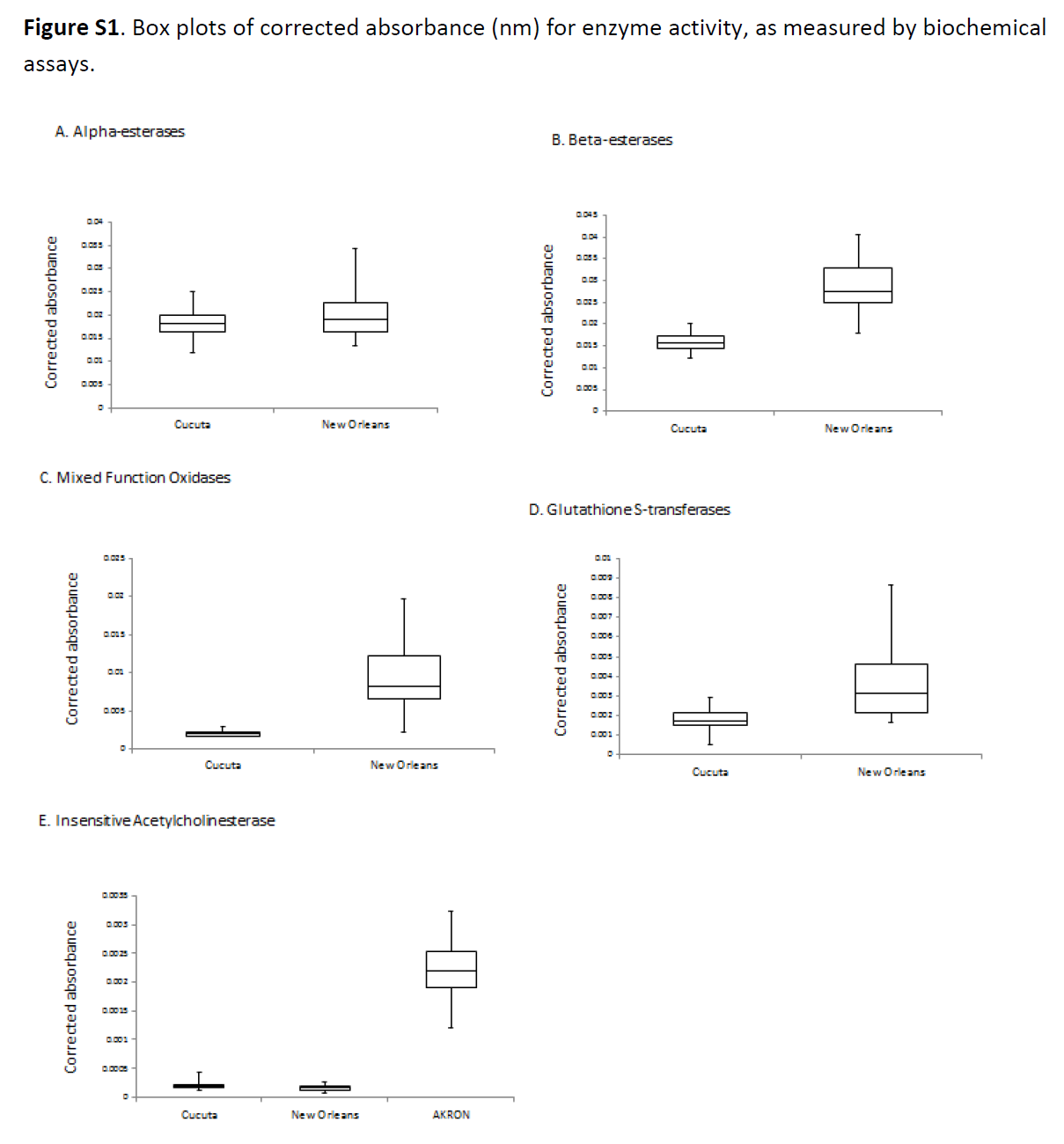

Supplement: Figure S1 — Box plots of corrected absorbance (nm) for enzyme activity, as measured by biochemical assays. A: Alpha-esterases; B: Beta-esterases; C: Mixed function oxidases; D: Glutathione S-transferases; E: Insensitive acetylcholinesterase. (TIF) [file pntd.0002438.s001.tif]
