# Supplementary material for: Temephos Resistance in Aedes aegypti in Colombia Compromises Dengue Vector Control
Source: PLoS Negl Trop Dis. 2013 Sep 19;7(9):e2438. doi: 10.1371/journal.pntd.0002438 (PMC3777894; doi:10.1371/journal.pntd.0002438)
Supplement: Table S1 — Summary of primer sequences for ace-1 PCR and sequencing. Ace1-For and Ace1-Rev were the external primers used in the PCR. All other primers were used only for sequencing. (RTF) [file pntd.0002438.s002.rtf]

Table S1. Summary of primer sequences for ace-1 PCR and sequencing. Ace1-For and Ace1-Rev were the external primers used in the PCR. All other primers were used only for sequencing.
Primer	Sequence	
Ace1-For	AGTGGAAAGAAAGTAGACGCATGGC	
Ace1-Rev	TAGAAGGGGTCGATAGGGCGTTGC	
Int_450-For	AACTTAGCATTGAGATGGGTACG	
Int_926-For	ATACTACTTGACTGAACTATTGCGG	
Int_1376-For	AAATCCAAGTCCTCCAAATAGTGAC	
Int_474-Rev	GCGTACCCATCTCAATGCTAA	
Int_951-Rev	CCGCAATAGTTCAGTCAAGT	
Int_1394-Rev	TTTGGAGGACTTGGATTTGG	
