# Supplementary material for: Temephos Resistance in Aedes aegypti in Colombia Compromises Dengue Vector Control
Source: PLoS Negl Trop Dis. 2013 Sep 19;7(9):e2438. doi: 10.1371/journal.pntd.0002438 (PMC3777894; doi:10.1371/journal.pntd.0002438)
Supplement: Table S2 — Summary of primer sequences for quantitative real-time PCR. (RTF) [file pntd.0002438.s003.rtf]

Table S2. Summary of primer sequences for quantitative real-time PCR
Primer	Sequence	Product length (bp)	
Ae60sL8_For	CTGAAGGGAACCGTCAAGCAA	119	
Ae60sL8_Rev	TCGGCGGCAATGAACAACT		
AeRPS7_For	GTTGGAGATGAACTCGGACCTG	87	
AeRPS7_Rev	GCCTTCTTGCTGTTGAACTCG		
AeCYP6N12_For	TGGGTGCTGTGAGGGATAC	122	
AeCYP6N12_Rev	AGTCAATGTCTCTGTGTTGCC		
AeCYP6F3_For	GCCGAGGTGGAGCAGTTC	170	
AeCYP6F3_Rev	TTCTTGGAATTGGTGGAGGTTTC		
Ae015010_For*	GGAAACTGGGGGACATGTGA	110	
Ae015010_Rev*	CACTTTCGTCCAGCAGGGTT		
*Primers for Acetyl CoA
